# Metabolic diversity and ecological niches of *Achromatium* populations revealed with single-cell genomic sequencing

**DOI:** 10.3389/fmicb.2015.00822

**Published:** 2015-08-10

**Authors:** Muammar Mansor, Trinity L. Hamilton, Matthew S. Fantle, Jennifer L. Macalady

**Affiliations:** ^1^Geosciences Department, Pennsylvania State UniversityUniversity Park, PA, USA; ^2^Department of Biological Sciences, University of CincinnatiCincinnati, OH, USA

**Keywords:** *Achromatium*, sulfur oxidation, Warm Mineral Springs, intracellular calcite, single-cell genomics, V-type ATPase, inclusion membrane proteins, carbonate precipitation

## Abstract

Large, sulfur-cycling, calcite-precipitating bacteria in the genus *Achromatium* represent a significant proportion of bacterial communities near sediment-water interfaces at sites throughout the world. Our understanding of their potentially crucial roles in calcium, carbon, sulfur, nitrogen, and iron cycling is limited because they have not been cultured or sequenced using environmental genomics approaches to date. We utilized single-cell genomic sequencing to obtain one incomplete and two nearly complete draft genomes for *Achromatium* collected at Warm Mineral Springs (WMS), FL. Based on 16S rRNA gene sequences, the three cells represent distinct and relatively distant *Achromatium* populations (91–92% identity). The draft genomes encode key genes involved in sulfur and hydrogen oxidation; oxygen, nitrogen and polysulfide respiration; carbon and nitrogen fixation; organic carbon assimilation and storage; chemotaxis; twitching motility; antibiotic resistance; and membrane transport. Known genes for iron and manganese energy metabolism were not detected. The presence of pyrophosphatase and vacuolar (V)-type ATPases, which are generally rare in bacterial genomes, suggests a role for these enzymes in calcium transport, proton pumping, and/or energy generation in the membranes of calcite-containing inclusions.

## Introduction

The genus *Achromatium* contains morphologically striking and unusually large bacteria distinguished by their abundant calcium carbonate inclusions. A single *Achromatium* cell may be shaped as a coccus or rod with a length between 10 and 100 μm, with large calcite inclusions (5–6 μm) and smaller sulfur inclusions (0.5–2 μm) (Gray and Head, 2014). Additional small inclusions containing elevated concentrations of Ca^2+^ ions (>1 mM) have recently been described in *Achromatium* populations from a salt marsh (Salman et al., 2015). *Achromatium* cells have been observed in surface sediments of both fresh and marine waters throughout the world and can reach population densities as high as 10^5^ cells/ml (Head et al., 1995, 1996; Gray and Head, 2014; Salman et al., 2015). Given the large size of *Achromatium* cells, at this cell density they can comprise over 90% of the bacterial biovolume within a few centimeters of the sediment-water interface (Head et al., 1995, 1996). Despite their large biomass and mysterious carbonate precipitation behavior, the role of *Achromatium* populations in biogeochemical cycling is not well understood. A differential sedimentation technique can be used to enrich for calcite-filled *Achromatium* cells based on their density (de Boer et al., 1971), but has yet to yield a pure culture.

There is strong evidence in the literature that *Achromatium* utilize reduced sulfur sources as electron donors (Gray et al., 1997, 1999b). In addition, *Achromatium* can fix organic carbon and assimilate acetate (Gray et al., 1999b, 2000), and can potentially use nitrate as an electron acceptor (Gray et al., 2004). *Achromatium* cells are capable of vertical migration by rolling, jerky motions, and their vertical migrations in sediment have been interpreted as a response to oxygen gradients (Gray et al., 1997). This apparent chemotaxis suggests that they use oxygen as an electron acceptor (Gray et al., 1997; La Riviere and Schmidt, 2006; Gray and Head, 2014). Under anoxic conditions, *Achromatium* have been hypothesized to gain energy from the reduction of nitrate, iron, manganese, or elemental sulfur (Gray et al., 1997; Head et al., 2000b; Gray and Head, 2014) although none of these activities have been demonstrated to date.

Similar to *Achromatium*, several strains of cyanobacteria are capable of precipitating intracellular calcite (Couradeau et al., 2012; Benzerara et al., 2014). Given the cosmopolitan distribution and early rise of cyanobacteria in Earth's history, this uncharacterized calcification pathway may have been integral to both the global calcium and carbon biogeochemical cycles on early Earth (Couradeau et al., 2012; Benzerara et al., 2014). In freshwater sediments from a wetland region close to Rydal Water in Cumbria, UK (54°27′N, 3°00′N), the solid-phase calcium content across sediment depths correlates with *Achromatium* cell numbers, indicating that intracellular, prokaryotic calcification plays an important role in controlling calcium and carbonate distributions in surface sediments (Head et al., 1995, 2000b).

The function of calcite inclusions in prokaryotes is not known. Current hypotheses include dissolution or precipitation to regulate acidity generated by sulfur oxidation (La Riviere and Schmidt, 2006; Salman et al., 2015), to generate protons to dissolve acid-volatile sulfides (AVS) (Gray, 2006), to maintain a high pCO_2_ for carbon fixation (Head et al., 2000b), as buoyancy regulators (Babenzien, 1991; Couradeau et al., 2012) and as electron acceptors in carbonate respiration (Babenzien, 1991). In *Achromatium* cells, TEM analysis suggests the presence of membranes surrounding the calcite inclusions, as well as electron-dense central areas interpreted as sites of crystal nucleation (Head et al., 2000b; Gray and Head, 2014). Because pure cultures of *Achromatium* are not available, it has proven difficult to characterize the biochemical pathway of intracellular calcite precipitation as well as to determine the biological role of calcite inclusions in *Achromatium*'s physiology.

Natural communities of *Achromatium* described to date are comprised of multiple phylotypes with 16S rRNA gene sequence identities ranging between 90 and 100% (Head et al., 1996; Glöckner et al., 1999; Gray et al., 1999a; Salman et al., 2015). Combined microautoradiography and fluorescence *in situ* hybridization (FISH) indicated that physiological differences in *Achromatium* communities are not related to carbon metabolism (Gray et al., 2000). However, different populations of *Achromatium* appear to respond differently to nitrate under anoxic conditions (Gray et al., 2004), suggesting that energy metabolisms could differ among co-existing populations.

Single-cell genomic sequencing offers a suitable method to reveal the genetic potential of *Achromatium* populations and to test how co-occurring *Achromatium* populations differ in their genetic potential. Femtograms of DNA from a single cell obtained from an environmental sample can be amplified by multiple displacement amplification (MDA) and sequenced, revealing the metabolic capacities of uncultured microorganisms (Mußmann et al., 2007; Chitsaz et al., 2011; Marshall et al., 2012). In this study, we utilized single-cell genomic sequencing on multiple *Achromatium* populations from a single environmental sample. The three draft genomes represent phylogenetically diverse phylotypes and offer a first glimpse of the molecular machinery present in this enigmatic genus.

## Materials and methods

### Site description, sample collection, and geochemistry

**Figure 1:**
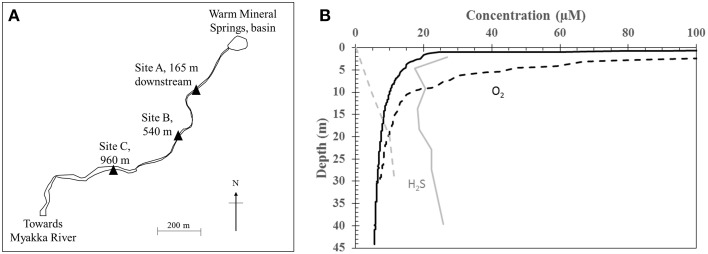
**(A)** Sampling sites at Warm Mineral Springs. **(B)** Water column depth profile of H_2_S (gray) and dissolved oxygen (black) within the basin. Solid and dashed lines indicate profiles for June 2012 and 28 October 2013 respectively.

Warm Mineral Springs (WMS) is a water-filled sinkhole located in the city of North Port, Florida, United States (27.06°N, 82.26°W). The spring consists of a central basin and a single outflow with tidal fluctuations up to 20 cm (Figure 1A). The spring basin is approximately 70 m deep and is fed by multiple warm springs that enter near the bottom. It has been popular as a spa since the 1960s due to its warm temperature of 30°C, a near-neutral pH of 7, and brackish composition equivalent to approximately 50% of seawater salinity. Human and animal remains and cultural artifacts are well preserved in the spring basin due to perennially low dissolved oxygen in the water column, making it an important archeological site (Clausen et al., 1975). A constant supply of dissolved sulfide in the water column, high sediment organic carbon content and nitrate levels of about 0.8 μM likely contribute to the diversity of sulfur-oxidizing bacteria found in the basin and spring outflow stream, including *Beggiatoa, Thiothrix, Thiovulum*, and *Achromatium* species described in reports since the early 1960s (Lackey and Lackey, 1961; Brigmon et al., 1994; Larkin et al., 1994). Bicarbonate concentration in the spring water is approximately 2 mM and elemental sulfur (S(0)) is present in the sandy sediments (Lackey and Lackey, 1961). Discharge from the basin is high (3.4 × 10^6^ L/day, Lackey and Lackey, 1961; Clausen et al., 1975) and flows into a narrow channel that runs approximately 1.3 km downstream before joining the Myakka River.

The top 2 cm of sediments at WMS were sampled for genetic, microbiological, chemical, and physical analyses. Samples were collected by scooping the sediments with pre-sterilized tubes and bottles. Sediments used for elemental analysis were frozen at −20°C after storage at 4°C for less than 1 month. After thawing, they were centrifuged at 8000x g for 10 min and the pore water was removed. Sediments were subsequently freeze-dried and powdered. Sediment organic matter content was measured with the Loss-On-Ignition (LOI) method by heating overnight at 750°C followed by further heating at 900°C for 3 h. Aliquots of the same sample were decarbonated in 1N HCl overnight and dried at 80°C prior to LOI as a control to determine the effect of possible carbonate particles on organic matter measurements. Solid-phase acid-volatile sulfides (AVS_*solid*_) were dissolved in 1.2 N HCl mixed with 1:100 volume of Ti(III)-citrate as a reducing agent. Ti(III)-citrate was added to prevent the formation of S(0) during acid dissolution (Rickard et al., 2006; Guilbaud et al., 2011). Released H_2_S_(*g*)_ was channeled to 0.2 M zinc-acetate trapping solution with continuous N_2_ flow for at least an hour. The reaction was allowed to proceed until no more H_2_S_(*g*)_ was produced, as determined by a PGD2 portable gas detector (ENMET Corp., Ann Arbor, MI, USA). Leakage was continuously monitored with the portable gas detector. Precipitated zinc sulfide minerals were subsequently dissolved and analyzed with the methylene blue method (Hach Co., Loveland, CO, USA) for AVS_*solid*_ measurements.

Dissolved oxygen (DO), pH, conductivity and temperature of the spring water were determined *in situ* in the WMS basin with a YSI 6600 multi-parameter sonde probe (YSI Incorporated, Yellow Springs, OH, USA) or in the WMS outflow streams using sensors attached to a 50i multimeter (WTW, Weilheim, Germany) calibrated daily with fresh standards. Dissolved sulfide, sulfate, nitrate, iron and ammonium concentrations were measured in the field on a portable spectrophotometer using the methylene blue, SulfaVer 4, Cadmium Reduction, FerroVer and Salicylate methods respectively (Hach Co., Loveland, CO, USA). Filtered (<0.2 μm) water samples were collected into acid-washed polypropylene bottles and tubes during sampling events in 2012 and 2013 to determine the concentrations of major cations and anions. Filtered water samples were immediately placed on ice and stored at 4°C until further processing. Cations were measured on acidified samples with Inductively Coupled Plasma-Atomic Emission Spectroscopy and anion concentrations were measured with a Dionex ICS-2500 Ion Chromatogram at Pennsylvania State University.

Based on our observations, *Achromatium* populations decline after 2 weeks in natural sediments stored at temperatures between 4 and 30°C. Thus, fresh sediments from the basin were regularly collected as described above and shipped on ice overnight to Pennsylvania State University for further processing.

### Cell purification

Fresh sediments were sequentially filtered through pre-sterilized 100, 63, and 40 μm sieves to remove particles larger than *Achromatium*. The cells were concentrated using the “gold panning” method that takes advantage of their fast sedimentation rate (de Boer et al., 1971; Glöckner et al., 1999) and counted by direct microscopic observation. After the heavy cells sedimented, the majority of small particles were carefully removed by repeated decanting in distilled water. Enriched *Achromatium* cells were then sorted by flow cytometry through a Beckman Coulter MoFlo Astrios employing a 100 μm nozzle and a flow rate of approximately 2000 events per second. This fraction was washed three times after sorting with UV-treated phosphate-buffered saline (PBS) at pH 7 to remove residual free DNA. Single cells of *Achromatium* were carefully hand-picked with a pre-sterilized syringe needle (127 μm inner diameter, Hamilton Company, Reno, NV, USA) attached to a micromanipulator, transferred to 3 μl of UV-treated PBS, and immediately frozen at −20°C.

### Genomic DNA amplification, sequencing, and assembly

Genomic amplification was carried out using the REPLI-g Single Cell Kit according to manufacturer's protocol (Qiagen, Valencia, CA). Briefly, single *Achromatium* cells in PBS were thawed at room temperature and lysed following the addition of alkaline Buffer D2 solution at 65°C for 10 min. Stop Solution was added to stop the lysis reaction and to neutralize the solution. Genomic DNA was then amplified with Φ DNA polymerase at 30°C for 8 h followed by deactivation at 65°C for 3 min. Results of the amplification were checked by NanoDrop (Thermo Fisher Scientific Inc., Wilmington, DE). Amplified genomic products were then screened for the presence of 16S rRNA gene sequences using 1 uL of 1:25 dilutions of amplified gDNA in a polymerase chain reaction (PCR) with 27f and 1492r primers (Lane, 1991). PCR cycling conditions were described previously (Macalady et al., 2008). Unpurified PCR products were sequenced by Sanger sequencing at the Genomics Core Facility at Pennsylvania State University. Partial sequences were assembled with CodonCode Aligner, version 4.0.4 and were manually checked for chimeras. Three amplified genomic products (WMS1, WMS2, and WMS3) contained single 16S rRNA gene sequences, and all were affiliated with the genus *Achromatium* based on BLASTN. The 16S rRNA gene sequences from the single cells and closely related sequences identified with BLASTN searches were automatically aligned to the SILVA reference alignment using the SINA Webaligner and merged into the SILVA version 108 database (Pruesse et al., 2007). The alignment was manually refined in ARB (Ludwig et al., 2004). To rigorously assess the placement of the single cell genomes in the genus *Achromatium* using the 16S rRNA sequences, multiple phylogenetic tree construction methods were employed. Neighbor-joining, maximum parsimony and maximum likelihood trees were calculated in ARB using 1000 bootstrap replicates. Trees were calculated in MEGA (ver. 6.05; Tamura et al., 2013) using neighbor-joining and maximum likelihood methods and the tree topology was evaluated by 1000 bootstrap resamplings. One thousand bootstrap samples were generated and evaluated in PhyML (version 2.4.1; Guindon and Gascuel, 2003) under the HKY85 substitution model and the gamma distribution with four rate categories. The relationship among *Achromatium* spp. was further examined by Bayesian phylogeny inference analyses with the HKY substitution matrix for 100,000 generations with MrBayes (Ronquist et al., 2012). All trees were congruent and displayed identical branching order. 16S rRNA secondary structures were visualized with the XRNA software (http://rna.ucsc.edu/rnacenter/xrna/xrna.html).

The three amplified genomic products were subjected to whole genome sequencing at the Genomics Core Facility at Pennsylvania State University. Genomic libraries were prepared using a TruSeq DNA PCR-Free kit (Illumina, San Diego, CA) to reduce bias and sequenced using the Illumina MiSeq platform (paired ends, 250 bp read length) (Illumina, San Diego, CA). Quality of the reads was checked with FastQC (bioinformatics.babraham.ac. uk/projects/fastqc). Based on the quality check, the first 10 and last 15 base pairs of the reads were trimmed with Trimmomatic (Bolger et al., 2014), with further trimming at the ends if the read qualities fell below a phred-33 quality value of 20. Only reads with a minimum length of 50 base pairs were used for assembly. Reads from each single cell (WMS1, WMS2, WMS3) were assembled independently using IDBA-UD (Peng et al., 2012), Velvet-sc (Chitsaz et al., 2011), and A5-MiSeq (Coil et al., 2014). The resulting assemblies were compared using Quast (Gurevich et al., 2013) and the best assembly for each cell was chosen based on the longest contig, the fewest number of contigs and the largest N50. Assembly with IDBA-UD was the best in all cases. To further improve assembly, a step size of two was chosen for k-mer increments up to 201 in IDBA-UD and the output was manually checked for the best assembly based on the criteria listed above. Contigs less than 500 bp were removed from each assembly. Each assembly was then assessed for the presence of contaminants using BLASTN. Contigs with high similarity to eukaryotic or phage sequences were removed. The potential presence of microbial contaminant sequences was evaluated through a binning approach within the program MetaWatt (version 1.7; Strous et al., 2012). Genome completeness was estimated based on a set of conserved, single-copy housekeeping genes (Supplementary Table 1; Gil et al., 2004).

### Annotation

The draft genomes were annotated using RAST (Aziz et al., 2008; Overbeek et al., 2014) and the Kyoto Encyclopedia of Genes and Genomes server (KEGG; Kanehisa and Goto, 2000). Annotation of respiratory genes was manually checked with BLASTP, with an *e*-value cutoff of <1 × 10^−4^ and a minimum identity coverage of 75%. An annotation was accepted as correct only if two out of the three methods described gave the same functional annotation. Amino acid sequences annotated as sulfide:quinone reductase (Sqr) and type 1 [NiFe]-hydrogenases from the single cell genomes were further grouped into their respective homologs based on phylogenetic analyses. The sequences were aligned with ClustalX. Alignments were populated with closely related sequences and manually curated. Phylogenetic trees were prepared using MEGA as described above. The Sqr sequences were classified according to Gregersen et al. (2011) while type 1 [NiFe]-hydrogenases were classified based on Pandelia et al. (2012). For identification of genes encoding enzymes necessary for nitrate respiration, full-length sequences of component proteins of Nap and Nar were mined from public databases using functional annotation and BLAST. Multiple query sequences for BLAST searches against the single cell genomes were chosen. To be identified as a putative Nap or Nar component, BLASTP/BLASTX hits were required to meet the following criteria: each functional gene for all alignments had to cover greater than 70% of the total query, with *e*-value scores lower than 1e-4 and a bit score >100. Putative calcium-binding motifs in amino acid sequences were identified via Prosite (prosite.expasy.org) based on a motif list prepared by Santamaría-Hernando et al. (2012). Three publicly available genomes of cyanobacteria that contain carbonate inclusions (Benzerara et al., 2014) were downloaded from NCBI for comparison. These consists of *Chroococcidiopsis thermalis* PCC 7203 (Shih et al., 2013), *Cyanothece* sp. PCC 7425 (Bandyopadhyay et al., 2011) and *Thermosynechococcus elongatus* BP-1 (Nakamura et al., 2002).

### Nucleotide sequence accession numbers

Raw sequence reads of all samples were deposited at the NCBI Short Read Archive (SRA) and can be accessed under the accession numbers SRR1771604, SRR1771715, and SRR1771809. Draft genome sequences were deposited at GenBank under the accession numbers JXSM00000000, JXSN00000000 and JXSO00000000 and the versions described in this paper are JXSM00000000, JXSN00000000, and JXSO00000000. 16S rRNA gene sequences have been deposited in GenBank under the accession numbers KP694216 through KP694218.

## Results

### Geochemistry

Geochemistry of the WMS spring water was analyzed during sampling trips in 2012 and 2013. In 2012, dissolved oxygen (DO) in the basin was depleted (<20 μM) below a water depth of 2 m, while total sulfide concentration varied between 20 and 30 μM throughout the water column (Figure 1B). In contrast, on 28 October 2013, there was no detectable sulfide at the water surface. DO decreased with depth to <20 μM at 10 m while sulfide increased with water depth within the basin, reaching 10 μM at 20 m. The following day, 8 μM sulfide was detected at the water surface (Table 1). Other geochemical parameters showed no significant variations with depth in the basin. Nitrate remained at less than 0.16 μM throughout the water column. Nitrite was below the detection limit (0.5 μM). Dissolved calcium concentration was 11.1 mM. Total dissolved Fe remained at less than 0.5 μM throughout the water column. Other geochemical parameters in the basin were similar to values reported by Lackey and Lackey (1961).

**Table 1:** **Summary of surface water geochemistry at Warm Mineral Spring**.

**Site and distance downstream**	**Basin**			**Outflow Site A, 165 m**			**Site B, 540 m**		**Site C, 960 m**	
Date	6/12	28/10/13	29/10/13	27/10/13	28/10/13	29/10/13	28/10/13	29/10/13	28/10/13	29/10/13
*Achromatium* present?	Yes, 200–500 cells/cm^3^			No			Yes, <100 cells/cm^3^		No	
pH	7.03	7.32	7.12	7.09	7.10	7.14	7.11	7.10	7.19	7.21
Temp (°C)	29.6	31.7	29.3	29.3	30.0	28.4	29.0	27.7	27.3	26.1
Conductivity (mS/cm)	28.9	32.6	30.5	29.3	29.5	29.8	29.7	30.4	27.5	28.3
DO^a^ (μM)	255	191	11	36	22	18	39	17	73	65
Dissolved sulfide (μM)	33.1	BDL^b^	8.0	0.2	BDL	1.1	BDL	BDL	BDL	0.3
Sulfate (mM)	17.7	12.1	15.0	12.5	14.2	11.7	11.3	12.9	10.4	13.3
Nitrate (μM)	0.16	<1.00	<1.00	<1.00	<1.00	<1.00	<1.00	<1.00	<1.00	<1.00
Fe (μM)^c^	0.5	BDL	BDL	BDL	0.9	0.7	BDL	0.7	0.7	0.9
Ca (mM)	–	11.1	–	–	–	–	–	–	–	–
K (mM)	–	2.8	–	–	–	–	–	–	–	–
Na (mM)	–	147	–	–	–	–	–	–	–	–
Mg (mM)	–	20.0	–	–	–	–	–	–	–	–
Cl- (mM)	–	239	–	–	–	–	–	–	–	–
NH_3_ (μM)	–	20.8	–	–	–	–	–	–	–	–

a *Dissolved oxygen*.

b *BDL, Below Detection Limit. Method detection limit for total sulfide is 0.16 μM*.

c *Method detection limit, total Fe = 0.4 μM*.

Three locations along the outflow stream were chosen as sample sites in addition to the basin (Figure 1A). All of the outflow sample sites had near-neutral pH, low levels of dissolved Fe (<0.9 μM), low levels of dissolved sulfide (<1.1 μM) and low levels of nitrate (<1 μM) (Table 1). Dissolved sulfate was lower in the outflow sites relative to the basin, with the lowest concentration measured at Site C (10.4–13.3 mM). Temperature and conductivity at Site C were measured at 26.1–27.3°C and 27.5–28.3 mS/cm respectively. These values were lower relative to other outflow sites, which had values comparable to the basin (27.7–30°C and 29.3–30.4 mS/cm respectively). Furthermore, DO at site C was measured at 65–73 μM and this was higher relative to Sites A and B (17–39 μM). Geochemistry of Site C, the farthest downstream site sampled, is likely influenced by freshwater inputs leading to relatively lower concentrations of sulfate, lower temperature, lower conductivity and higher amounts of DO.

Surface sediments from all sampling sites were composed of organic-rich peat mixed with silicate sand. Surface sediments from the littoral zone of the basin were characterized by high porosity (0.9, *n* = 2), high organic matter (21.9–26.6 wt%, *n* = 2) and low AVS_solid_ (0.3–0.6 μmol/g solid, *n* = 3). Decarbonation prior to LOI analysis did not yield notably different estimates of sedimentary organic matter content, suggesting that inorganic carbon in carbonate minerals was not present in the sediment in significant amounts.

**Figure 2:**
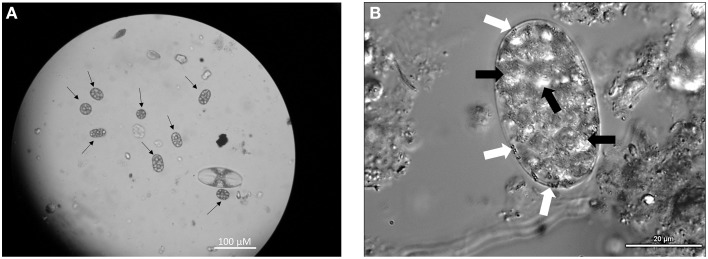
**(A)** Light microscopy image of *Achromatium* (black arrows), highlighting their range of sizes and shapes at Warm Mineral Springs. **(B)** Phase contrast microscopy of *Achromatium*. Calcite inclusions (black arrows) can be seen throughout the cell, while small sulfur inclusions (white arrows) can be seen at the periphery.

*Achromatium* cells were abundant in surface sediments in the littoral zone of the basin, reaching cell densities of 200–500 cells/cm^3^ of wet sediment. The size and shape of observed cells varied but typically exceeded 15 μm on the shortest axis with a maximum of 45 μm on the longest axis (Figure 2). Calcite and sulfur inclusions had diameters of 3–7 and 0.5–1.5 μm respectively. No white mats or veils typical of sulfur-oxidizing bacteria were observed at the sediment-water interface in the basin. Along the outflow channel, *Achromatium* was not observed at Site A or Site C where white microbial mats were abundant, but *Achromatium* was observed in the surface sediments from Site B where white microbial mats were absent or weakly developed. The abundance of *Achromatium* at Site B was <100 cells/cm^3^ of wet sediment.

### Cell isolation and phylogeny of *achromatium* populations

**Figure 3:**
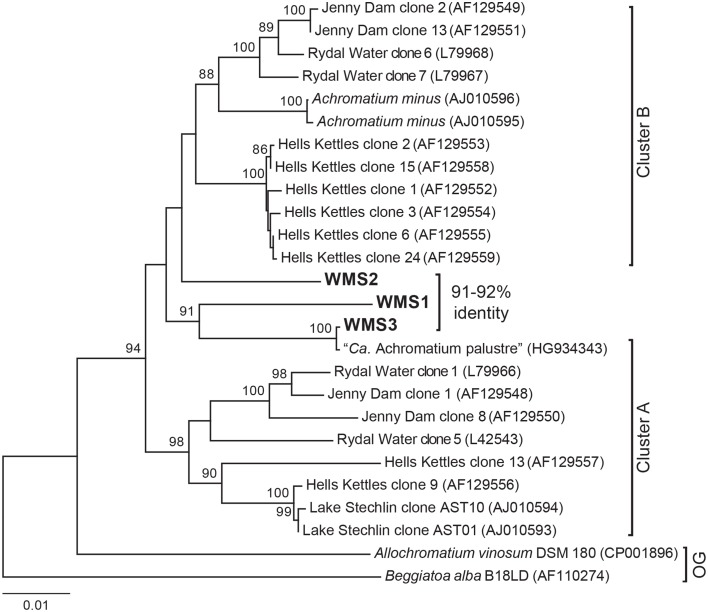
**Maximum likelihood tree of 16S rRNA gene sequences of ***Achromatium*** populations with ***Allochromatium vinosum*** and ***Beggiatoa alba*** as outgroups (OG)**. NCBI accession numbers are given in parentheses. Sequences from the single cells analyzed in this study were obtained by PCR of the amplified gDNA (WMS1, WMS2, and WMS3) and are identical to the 16S rRNA gene sequences recovered from the assembled genomic data for each cell. The percent identity of the WMS *Achromatium* 16S rRNA sequences relative to one another are highlighted. Bootsrap values >85 based on 1000 samplings are given for each node.

Single cells of *Achromatium* from a bulk sediment sample collected from the basin were isolated by a combination of differential sedimentation, flow cytometry, and micromanipulation. The sorted fraction from flow cytometry (Supplementary Figure 1) contained less than 4% *Achromatium* intermingled with abundant mineral particles and a few diatoms. Attempts to further purify the *Achromatium* cells based on size or lack of fluorescence were unsuccessful. Hand-picked single cells were subjected to whole genome amplification and screened for 16S rRNA genes prior to genomic sequencing. The WMS single cell 16S rRNA gene sequences had high sequence identity to known *Achromatium* phylotypes. WMS3 was most similar to “*Candidatus* Achromatium palustre” recovered from Sippewissett Salt Marsh (HG934343) with 99% 16S rRNA gene identity, while WMS1 and WMS2 were only weakly affiliated with existing clades (Figure 3). Based on visualization of 16S rRNA secondary structure, WMS3 has a deletion affecting helix 38 in the V6 region (“Cluster A”), whereas WMS1 and WMS2 lack the deletion (“Cluster B”) (Glöckner et al., 1999; Gray et al., 1999a; Salman et al., 2015).

### Genome completeness and purity

Single-cell genomes were sequenced with Illumina MiSeq, yielding 1.6–2.4 Gb of raw sequence data per genome. After removing low quality reads, over 99.5% of total reads remained. Assembly yielded draft genome sizes of 1.3, 2.8, and 3.8 Mbp for WMS1, WMS2, and WMS3 respectively (Table 2). Each draft genome sequence contained approximately 1000 contigs with N50 values ranging from 2561 bp for WMS1 to 8360 bp for WMS2. Based on single copy marker genes (Supplementary Table 1), WMS1 is 40% complete and WMS2 and WMS3 are both 80% complete. Estimated *Achromatium* genome sizes range between 3.3 and 4.8 Mbp.

**Table 2:** **Statistics for final draft genomes of ***Achromatium*** single cells after removal of contaminant sequences with high homology to ***Homo sapiens*** and bacteriophage ***phi*** X**.

**Statistics**
	**Cells**		
	**WMS1**	**WMS2**	**WMS3**
Raw data (Gb)	2.3	1.6	2.4
GC (%)	39	43	38
N50 (bp)	2561	8360	6610
Longest contig (bp)	12,446	47,002	31,024
# of contigs	669	777	1113
Average coverage	1000x	600x	600x
Assembly size (Mbp)	1.3	2.8	3.8
Genome completeness	40%	80%	80%
Estimated genome size (Mbp)	3.3	3.5	4.8

Using BLASTN, we identified several contigs with homology to *Homo sapiens* or the bacteriophage *phi* X. These sequences made up <0.1 to <0.3 Mbp of the total assembled sequences in each draft single cell genome. Contaminant sequences were removed immediately after assembly. We then used an intrinsic binning-based approach to identify potential microbial contaminants. Using MetaWatt's low confidence threshold (Strous et al., 2012), tetranucleotide binning grouped >98% of contigs from each draft genome into a single “Proteobacteria” bin. Microscopy observations prior to single-cell micromanipulation did not reveal any prokaryotic cells, and no growth was observed in mineral media amended with acetate. PCR with bacterial domain-specific primers 27f and 1492r yielded a single 16S rRNA gene sequence per cell. Finally, sequences of the assembled contigs within each draft genome displayed a unimodal distribution for GC%. Collectively, these data indicate no evidence for contamination in the draft genomes.

### Anabolism and motility

Each of the three draft genomes contains a partial set of the genes required for glycolysis, the tricarboxylic acid cycle and the synthesis of key amino acids and vitamins. Collectively however, the draft genomes contain the complete set of genes needed for glycolysis and the tricarboxylic acid cycle as well as for the synthesis of the 20 common amino acids and key vitamins. WMS3 also contained genes encoding for resistance to the antibiotics vancomycin (*vanW*) and penicillin (outer membrane protein gene *tolC*).

**Figure 4:**
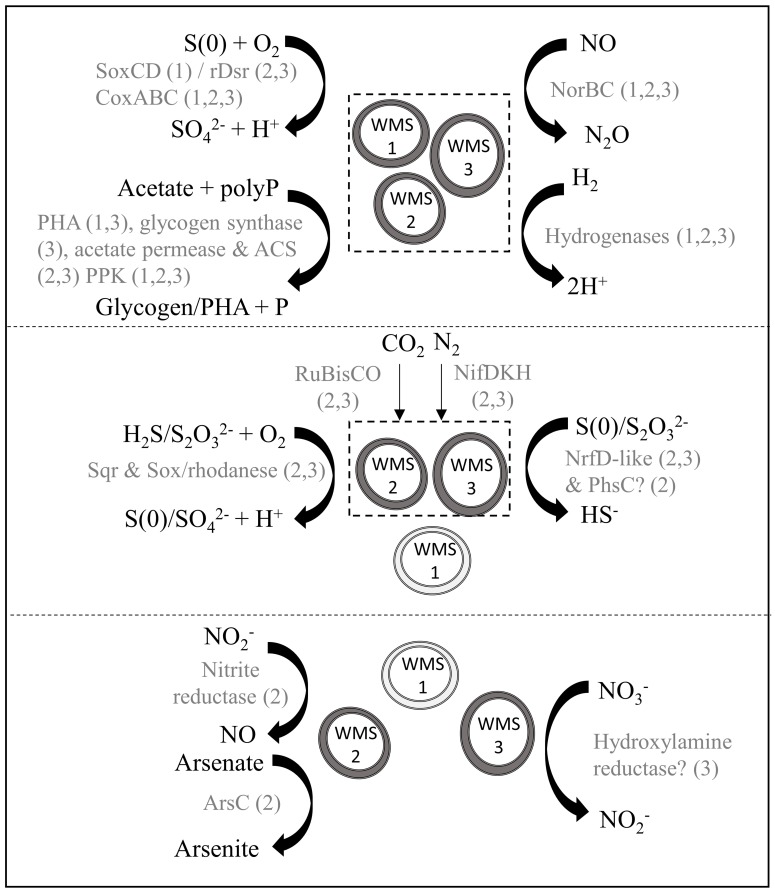
**Model of putative biogeochemical roles of ***Achromatium*** populations**. Different size/shape of *Achromatium* symbols represent different populations. Light gray *Achromatium* symbols indicate the genes were not identified in the draft genome, possibly due to amplification bias. Metabolic reactions are grouped according to those with genes identified in all three draft genomes **(top)**, in WMS2 and WMS3 **(middle)** and in a single draft genome **(bottom)**. Text in gray indicate the proteins catalyzing the reaction, with numbers in parentheses indicating a draft genome.

WMS2 and WMS3 had genes encoding for acetate permease, acetyl-CoA synthetase and RuBisCO (Figure 4; Supplementary Table 2), supporting previous observations that some *Achromatium* populations exhibit mixotrophic or facultative lithoautotrophic lifestyles (Gray et al., 1999b, 2000). No evidence for known carbon fixation pathways other than the Calvin-Benson-Bassham Cycle was detected. Carbonic anhydrase was detected in all three draft genomes. WMS2 and WMS3 encoded the nitrogen fixation structural component gene *nifDHK*. Genes encoding for glutamate dehydrogenase, required for ammonium incorporation, were also detected in all three draft genomes.

The genes enabling *Achromatium*'s jerky, rolling motility potentially belong to the twitching motility gene set (*pil*). The WMS3 draft genome contained the complete *pil* gene set (*pilGHIJTU*) while the WMS1 and WMS2 draft genomes contained at least one gene involved in twitching motility. Several genes encoding chemotaxis response proteins were identified including the aerotaxis receptor (*aer*), the *cheABRWY* complex, motility protein B (*motB*) and genes encoding for various methyl-accepting chemotaxis proteins. The regulators of these genes/proteins are not known (Marshall et al., 2012). However, the presence of these genes is consistent with previous observations of vertical migration of *Achromatium* cells within sediment columns hosting chemical gradients (Gray et al., 1997).

### Energy metabolism

We identified multiple genes encoding enzymes involved in sulfur oxidation in the *Achromatium* draft genomes. Sulfide may be converted to S(0) through the activity of sulfide:quinone reductase (Sqr). A gene encoding for Sqr type C was identified in WMS2 while a gene encoding Sqr type A was identified in WMS3 (Supplementary Figure 2). The presence of at least one gene in the Sox pathway suggests that populations represented by WMS2 and WMS3 can convert thiosulfate to S(0)/sulfate. S(0) may be further converted to sulfite via rDsrAB or SoxCD (Loy et al., 2009; Gregersen et al., 2011). WMS2 had the genes encoding both subunits of rDsrAB while only the gene encoding rDsrB was identified in WMS3. The genes encoding SoxCD were not identified in WMS2 or WMS3. In contrast, a sequence encoding SoxD was identified in WMS1. Neither genes encoding for SoxC nor rDsrAB were present in WMS1. In addition, genes encoding for rhodanese were detected in WMS2 and WMS3. Rhodanese may function to oxidize thiosulfate to form sulfite, sulfate or S(0) (Smith and Lascelles, 1966; Schedel and Truper, 1980). Conversion of sulfite to sulfate may be catalyzed by genes encoding for sulfate adenyltransferase (*sat*), adenosine phosphosulfate reductase (*aprAB*) and heterodisulfide reductase (*hdrABC*) (Mußmann et al., 2007; Gregersen et al., 2011), which are partially/completely present in the draft genomes.

All three draft genomes contained genes encoding putative proteins with sequences homologous to hydrogenases. The draft genome of WMS2 encodes an Isp type 1 [NiFe]-hydrogenase (Supplementary Figure 3) while WMS1 and WMS3 have genes encoding for [FeFe]-hydrogenases with distant homology to coenzyme F420 oxidoreductase. WMS2 and WMS3 also have genes that encode an additional [FeFe]-hydrogenase.

We retrieved at least one gene encoding for components of cytochrome *c* oxidase (*coxABC*) in each single-cell genome, suggesting that all three populations have the potential to utilize oxygen as an electron acceptor. We did not identify any genes that encode typical nitrate reductase enzymes (i.e., Nar, Nap). In WMS3, we found a gene encoding for hydroxylamine reductase, which may function in dissimilatory nitrate reduction to ammonium (Jones et al., 2015). WMS2 can potentially reduce nitrite to nitric oxide via a copper-containing nitrite reductase (EC 1.7.2.1). rDsrAB, encoded in the draft genome of WMS2, is also known to slowly reduce nitrite to ammonia (Haveman et al., 2004). All three draft genomes include *norBC*, which encode the machinery necessary to reduce nitric oxide to nitrous oxide (e.g., Watmough et al., 1999).

In addition to oxygen, nitrite and nitric oxide, several other electron acceptors are potentially utilized by *Achromatium* based on annotated genes in the draft genomes, including polysulfide, thiosulfate and arsenate. In WMS2 and WMS3 we identified protein coding genes with homology to the NrfD-like polysulfide reductase, which catalyzes the reduction of polysulfide to sulfide (e.g., Krafft et al., 1995). In WMS2 we also identified a gene that encodes the C subunit of PhsABC, which functions as a thiosulfate reductase (Mußmann et al., 2007). A gene encoding an arsenate reductase (*arsC*) was identified in WMS2. No genes encoding proteins implicated in Fe reduction were identified. Siderophore biosynthesis genes were similarly not identified, even though genes encoding for TonB-dependent receptors and siderophore-specific ABC transporters were present in the draft genomes.

Additionally, we identified multiple genes related to diverse inclusions in *Achromatium*. Genes encoding for sulfur globule protein B (SgpB) (Brune, 1995) were identified in WMS2 and WMS3. We also identified genes encoding a polyphosphate kinase (PPK) in all three draft genomes, suggesting that *Achromatium* can utilize polyphosphates (polyP) and may store them for energy reserves. However, PPK alone does not indicate the presence of polyP granules. Finally, we identified genes encoding for polyhydroalkanoate synthase (PHA) in WMS1 and WMS3 and for glycogen synthase in WMS3. Acetate may be converted to either PHA or glycogen for carbon and energy storage using energy gained from polyP degradation, similar to *Thiomargarita* (Schulz and Schulz, 2005) and marine *Beggiatoa* (Mußmann et al., 2007).

### Possible calcification-related genes

Carbonic anhydrase, which interconverts bicarbonate (${\text{HCO}}_{3}^{-}$) and CO_2(*aq*)_, was identified in all three draft *Achromatium* genomes. Genes encoding for Ca^2+^-ATPase (PMCA-like membrane pump) and a predicted Na/H^+^-Ca exchanger (*yrbG*) were identified in WMS2 and WMS3. We also identified several genes encoding subunits of vacuolar(V)-type ATPases and for pyrophosphatase (PPase), which can generate a transmembrane H^+^ electrochemical gradient that can be used to drive transport of substrates across biological membranes (Martinoia et al., 2000; Beyenbach and Wieczorek, 2006).

Predicted proteins in the *Achromatium* draft genomes were examined to identify Ca-binding motifs, which could participate in calcification reactions. Several homologous proteins containing cadherin and hemolysin motifs were identified in WMS1 and WMS3 but not in WMS2. All of these proteins have unknown functions based on annotations. Other Ca-binding proteins identified in *Achromatium* were annotated as enzymes with caspase activity, NADH activity or involved in sugar biosynthesis.

We compared the *Achromatium* genes to genes present in the genomes of three cyanobacteria capable of forming calcite inclusions (Benzerara et al., 2014). Carbonic anhydrase genes were present in *C. thermalis* PCC 7203 and *Cyanothece* sp. PCC 7425 but not in *T. elongatus* BP-1. Genes encoding for Ca^2+^-ATPase were present in all three cyanobacteria while genes encoding for other membrane transporters (Na/H^+^-Ca^2+^ exchanger, V-type ATPases and PPase) were missing. *Achromatium* proteins containing hemolysin motifs were very distantly related (<30% identity) to Ca-binding proteins in the genomes of *C. thermalis* PCC 7203 and *Cyanothece* sp. PCC 7425 and had no homology to proteins in the genome of *T. elongatus* BP-1. Proteins containing cadherin motifs in *Achromatium* did not share any homology to proteins in the three cyanobacteria.

## Discussion

### Phylogeny

*Achromatium* phylotypes have been grouped into two clusters based on phylogenetic analysis of the 16S rRNA gene and the presence/absence of a deletion affecting helix 38 in the V6 region (Glöckner et al., 1999; Gray et al., 1999a; Salman et al., 2015). However, the WMS phylotypes are deeply branching sister groups to preexisting clades and do not fall neatly within existing taxonomic clusters based on phylogenetic analyses of full-length 16S rRNA genes carried out using multiple tree-building methods (Figure 3). WMS3 is closely related to the only known marine representative, “*Ca*. Achromatium palustre.” Both WMS3 and “*Ca*. Achromatium palustre” lack helix 38, but are not affiliated with other Cluster A sequences. WMS1 and WMS2 both have intact helix 38 but are only weakly affiliated with each other or with other Cluster B phylotypes.

### Habitats of *achromatium*

*Achromatium* populations have been found in freshwater sediments that are oxic or microoxic with low levels of sulfide (<30 μM), and in salt marsh sediments where sulfide concentrations are highly variable (0–2500 μM). The freshwater sediments are characterized by a weak sulfide generation capacity (low sulfate reduction rates) and/or continuous sulfide removal through iron sulfide precipitation (Babenzien, 1991; Babenzien and Sass, 1996; Head et al., 2000b; Gray, 2006; Gray and Head, 2014), leading to the suggestion that *Achromatium* cells may benefit from the ability to access reduced sulfur trapped in minerals such as iron sulfides (Gray, 2006; Gray and Head, 2014). Previous studies have shown that microbial oxidation of sulfide minerals to S(0)/sulfate can be coupled to nitrate, nitrite and/or oxygen reduction at neutral pH (e.g., Drobner et al., 1992; Vaclavkova et al., 2014 and references therein), although the mechanisms for accessing the sulfide at neutral pH are not well-studied. Our data suggest that dissolved sulfide may be continuously or intermittently limiting in the shallow-water, sunlit surface sediments at WMS. *Achromatium* is abundant at outflow Site B, where dissolved sulfide in the bulk water was below detection limits (<0.16 μM) at the time of sampling, and in the littoral zone of the basin, where sulfide concentrations were highly variable with a maximum of 30 μM over a 24 h period.

To evaluate whether *Achromatium* in the WMS basin sediments could benefit by accessing sulfide minerals for reducing power, we compared the amount of total dissolved sulfide available in the sediment pore water (assuming 30 μM concentration) vs. the amount of sulfide trapped in sulfide minerals (AVS_solid_) over the same sediment depth interval. Accounting for porosity, 1 cm^3^ of sediment contains approximately 0.03 μmol of dissolved sulfide and 0.05–0.10 μmol of sulfide trapped in sulfide minerals. Therefore, *Achromatium* would gain an advantage relative to other sulfur-oxidizing bacteria in the surface sediments by being able to access sulfide from AVS_solid_.

Relatively high sediment organic carbon content has been postulated as another factor that defines the ecological niche of *Achromatium* (Head et al., 2000a,b) and indeed sediments at WMS contain a high concentration of organic matter, similar to other habitats where *Achromatium* is abundant (Table 3). Based on the presence of RuBisCO, acetate permease and acetyl-CoA synthetase genes in the WMS2 and WMS3 draft genomes, *Achromatium* populations at WMS can likely both assimilate organic carbon and fix inorganic carbon, similar to the well-studied *Achromatium* populations at Rydal Water (Gray et al., 2000). In contrast, *Achromatium* populations in a freshwater pond near Hell Kettles, County Durham, UK, (54°29′N, 1°33′W) do not appear to contain genes for RuBisCO, but can assimilate organic carbon (Gray et al., 2000). Heterotrophy may thus be a conserved trait within the *Achromatium* genus, whereas autotrophy is not.

**Table 3:** **Organic content of sediments harboring ***Achromatium*****.

**Location**	**Org matter (wt%)**	**Org C (wt%)**
Warm Mineral Springs^a^	21.9–22.6	–
Rydal Water^b^	–	14.8–15.9
Hell Kettles^b^	–	21.3–22.2
Jenny Dam^b^	–	13.4–22.0
Sippewissett^c^	50–80	–
Lake Stechlin^d^	53	–
Lake Dagow^e^	–	18–21
Lake Fuchskuhle^e^	–	43–44

a *This study*.

b *Head et al. (2000b)*.

c *Howarth and Giblin (1983)*.

d *Casper (1985)*.

e *Measured as weight % organic matter by the Loss-On-Ignition method, but reported as weight % organic carbon. Conversion factor from organic matter to organic carbon was not reported. Data for Lake Dagow and Lake Fuchskuhle are from Conrad et al. (2009) and Conrad et al. (2010), respectively*.

Water flow characteristics including turbulence have been shown to be important in predicting the outcome of competition among sulfur-oxidizing *Beggiatoa, Thiothrix* and Sulfurovumales-group Epsilonproteobacterial populations in sulfidic cave waters (Macalady et al., 2008). This is not surprising since flow characteristics directly influence gas exchange and water chemistry as well as forces that can sweep away non-attached cells. In the sample locations where we detected *Achromatium* (basin and outflow site B), we observed little turbulence and a lack of conspicuous mat-forming sulfur oxidizers. *Achromatium* was not detected at sample sites in between or further downstream, where water turbulence was consistently higher. Our observations are consistent with the idea that water flow characteristics play an important role in controlling the distributions of *Achromatium*.

### Metabolic versatility of *achromatium* populations

Based on the draft genomes, *Achromatium* populations at WMS are likely carbon mixotrophs with highly versatile respiratory metabolisms. WMS populations can probably generate energy from the oxidation of sulfide, thiosulfate, S(0), and hydrogen. Interestingly, phylogenetically distinct *Achromatium* cells had significant differences in their genetic potential for carrying out energy generation reactions (Figure 4; Supplementary Table 2). In terms of sulfide oxidation, SqrA (encoded in the WMS3 genome) is well characterized and has a high (micromolar) affinity for sulfide (Marcia et al., 2010), while the sulfide affinity of SqrC (encoded in the WMS2 genome) is not well known. WMS2 and WMS3 appear to use rDsrAB for S(0) oxidation, while WMS1 uses SoxCD. rDsrAB and SoxCD genes are typically not found in the same organism (Loy et al., 2009; Gregersen et al., 2011), with the only known exception in *Roseobacter* (Lenk et al., 2012). Genes encoding three types of hydrogenases were identified in the draft genomes. One of them, Isp type 1 [NiFe]-hydrogenase, has been linked to sulfur-dependent metabolism (Pandelia et al., 2012). Type 1 [NiFe]-hydrogenases are the primary hydrogenases for growth on H_2_ coupled to reduction of O_2_, nitrate, sulfate, S(0), fumarate or CO_2_, and also recycle H_2_ produced by the enzyme nitrogenase (Vignais et al., 2001). The other two hydrogenases we identified are not closely affiliated to any well-characterized hydrogenases, and their exact functions are therefore unknown. In general, hydrogenases are oxygen sensitive although several forms maintain catalytic activity in oxygenated environments (Vignais and Billoud, 2007; Pandelia et al., 2012). In *Achromatium*, it is possible that different types of hydrogenases may be better suited to different redox zones in the sediments due to varying levels of oxygen.

We have strong evidence based on the presence of terminal oxidases and other respiratory genes that *Achromatium* populations at WMS can utilize oxygen, polysulfide/thiosulfate, and NO as electron acceptors. We found only weak evidence for nitrate and nitrite respiration genes. The WMS3 draft genome contains a gene encoding a putative nitrate reductase (hydroxylamine reductase). The WMS2 draft genome contains a gene encoding a copper-containing nitrite reductase (EC 1.7.2.1). Genes encoding typical nitrate reductase enzymes (Nap, Nar) were not detected even after an extensive BLASTP/BLASTX search of the *Achromatium* draft genomes (assembled and unassembled reads) against sequences of well-characterized nitrogen respiration enzymes. Although nitrate profiles within sediments showed no clear relationship with *Achromatium* cell numbers in one previous study (Head et al., 2000a), Gray et al. (2004) showed that certain *Achromatium* populations survive longer under anoxic conditions when nitrate is provided. It is possible that nitrate and/or nitrite respiration genes are present in some or all *Achromatium* populations but were not observed due to amplification bias or incomplete coverage in the single-cell genomes.

### Genetic insights on mechanisms of calcite precipitation

Bicarbonate (${\text{HCO}}_{3}^{-}$) is freely available in natural waters and can be interconverted to CO_2(*aq*)_ by the action of carbonic anhydrase, which is present in all three draft genomes. Carbonic anhydrase is an important enzyme in microbially mediated extracellular calcite precipitation (e.g., Kupriyanova et al., 2007; Li et al., 2010) and eukaryotic biomineralization (e.g., Mann et al., 2012; Marie et al., 2012), and could therefore be involved in modifying saturation states of calcite in *Achromatium* vacuoles.

Proteins containing Ca-binding motifs or Ca^2+^-channels could potentially participate in calcite biomineralization reactions, but we did not identify any putative calcification genes by homology within the draft genomes. Calcium concentrations in the cytoplasm and vacuoles could be regulated via membrane pumps such as Ca^2+^-ATPase (PMCA-like), Na/H^+^-Ca exchangers, V-type ATPases, and PPase. V-type ATPases are common in eukaryotes and archaea but are rarely present in bacteria (Beyenbach and Wieczorek, 2006). “*Candidatus* Allobeggiatoa,” which contains nitrate storage vacuoles, employs V-type ATPases and PPase to generate ATP by pumping protons into the cytoplasm across the vacuolar membranes (Beutler et al., 2012). Alternatively, proton gradients generated by V-type ATPases and PPase at the expense of ATP can be used to drive transport of substrates across vacuolar membranes (Martinoia et al., 2000; Beyenbach and Wieczorek, 2006) and were proposed to facilitate nitrate uptake in marine *Beggiatoa* (Mußmann et al., 2007). In *Achromatium*, V-type ATPases and PPase might be used to drive the uptake of Ca^2+^ from the cytoplasm into the vacuoles, or may have a direct role in energy metabolism as in “*Candidatus* Allobeggiatoa.” Genes encoding for the subunits of V-type ATPases and PPase in the *Achromatium* draft genomes reside on short contigs and no co-localized transporter genes were identified on these contigs. Thus, the function of these membrane transporters in *Achromatium* remains a subject for further investigation.

We compared the draft genomes of *Achromatium* to those belonging to three cyanobacterial species containing carbonate inclusions in hope of finding closely related genes that may participate in intracellular calcification. Out of all the possible calcification-related genes discussed, only Ca^2+^-ATPases are encoded in *Achromatium* (WMS2 and WMS3) and all three cyanobacterial genomes. Interestingly, V-type ATPases and PPase were not present in the genomes of these cyanobacteria. This suggests that either different calcification pathways are utilized in these different lineages or that the genes discussed here are not essential for intracellular calcification. Further studies are required to resolve this matter.

### Metabolic role of calcite inclusions

Carbonate inclusions are rare in prokaryotic cells and are a defining feature of the morphology of *Achromatium* as well as several species of cyanobacteria. Whereas cyanobacteria are known to induce the extracellular precipitation of calcium carbonate minerals as a result of rapid uptake of inorganic carbon during photosynthesis, bacteria such as *Achromatium* oxidize reduced sulfur compounds completely to sulfate, resulting in a net production of protons that should have a negative influence on carbonate precipitation. The function of intracellular carbonate inclusions in prokaryotes is not well understood, and may differ even among cyanobacterial species (Benzerara et al., 2014). Multiple hypotheses have been proposed for the function of carbonate inclusions in *Achromatium* (summarized in Gray and Head, 2014), and these are discussed briefly below in light of new data generated in our study.

Since we did not identify any genes involved in methanogenesis or acetogenesis in the three draft genomes, we consider it very unlikely that the carbonate in calcite serves as an unconventional electron acceptor in CO_2_ respiration (Babenzien, 1991). Head et al. (1995, 2000b) suggested that calcite precipitation could be used to maintain a high intracellular partial pressure of CO_2_ for carbon fixation. In the WMS basin water, we calculated a dissolved CO_2_ concentration of about 200 μM after accounting for salinity, pH and temperature (Fantle, 2010). This concentration is considerably higher than the dissolved CO_2_ concentration in marine water, which falls between 10 and 80 uM. Furthermore, whereas calcite inclusions are observed in all members of the genus to date, some *Achromatium* do not fix carbon and/or lack genes encoding RuBisCO (Gray et al., 1999b, 2000). Thus, inorganic carbon storage for carbon fixation cannot explain the presence of carbonate mineral inclusions in *Achromatium* populations.

Babenzien (1991) proposed that intracellular calcite precipitation is a buoyancy regulator to assist migration of cells along vertical sediment redox gradients. In this scenario, calcite-filled *Achromatium* cells sink deeper into the sediments, with subsequent dissolution to rise back to the surface. Contrary to what this hypothesis would predict, several studies have shown that cells at the surface have more calcite compared to cells deeper in the sediments (Babenzien, 1991; Head et al., 1995; Salman et al., 2015). Furthermore, several studies report that *Achromatium* cells are motile. We observed twitching motility in all populations at WMS, and at least one gene in the gene set coding for twitching motility was present in all three single-cell genomes. Salman et al. (2015) considered a modified version of this hypothesis in which *Achromatium* at the sediment-water interface benefit from the anchoring effect of dense inclusions. At WMS, we observed that *Achromatium* were abundant only in relatively calm niches and were outcompeted by filamentous giant sulfur oxidizers with holdfasts in more turbulent areas. Thus, high density may well prove to be an advantage since ballasted cells are less likely to be swept away. A similar role for carbonate mineral inclusions as ballast has been suggested for certain cyanobacteria (Couradeau et al., 2012).

Two remaining previously proposed hypotheses are nominally consistent with our data and with the limited literature on *Achromatium* physiology and ecology to date. Gray (2006) suggests that calcite precipitation provides a source of protons to solubilize AVS minerals under sulfide-limiting conditions. This hypothesis is consistent with the observation that freshwater *Achromatium* habitats are characterized by low and/or highly variable dissolved sulfide concentrations. At WMS, sulfide in AVS probably represents a larger reservoir of electron donor than total dissolved sulfide. AVS could thus serve as an external solid source of electron donor in addition to intracellular stored S(0). Mechanisms by which *Achromatium* could enhance AVS mineral dissolution include the production of siderophores (Page and Huyer, 1984) and excretion of protons from sulfur oxidation or calcite precipitation (Gray, 2006). The absence of siderophore synthesis genes but presence of siderophore transport genes in the draft genomes is difficult to interpret but does not preclude the possibility that proton-mediated dissolution may enhance AVS solubilization by *Achromatium*. We note however that calcite-filled *Achromatium* have been recently reported in a marine environment with high dissolved sulfide, where AVS may represent a much smaller resource (Salman et al., 2015).

Lastly, several authors suggest that calcite dissolution could function as a buffer against acidity generated by S(0) oxidation (La Riviere and Schmidt, 2006; Salman et al., 2015). Although it is unclear why such a buffer should be needed by *Achromatium* cells and not by other giant sulfur oxidizers found in geochemically similar habitats, recent work by Salman et al. (2015) documents correlations among internal cell Ca:S ratios, pH, dissolved sulfide, and dissolved oxygen. They provide clear evidence that the growth and consumption of calcite inclusions are linked to dynamic temporal and spatial changes in environmental conditions that influence both acid production and the availability of electron donors and acceptors. In their conceptual model, acid consumption during sulfide oxidation to stored S(0) proceeds with CaCO_3_ precipitation under sulfidic conditions, with subsequent dissolution of the calcite inclusions as acid is produced during S(0) oxidation to sulfate. Given the significant variations in the number of protons produced or consumed during each phase of respiration depending on whether O_2_ or oxidized nitrogen species are used as electron acceptors (Salman et al., 2015), Ca:S ratios would be expected to vary in a potentially complex fashion.

An additional hypothesis for the function of calcite inclusions emerges from our genomic data. Based on the recognition that calcite precipitation generates protons at circumneutral cytoplasmic pH (Equation 1):

(1) $$\begin{array}{r} \text{C}{\text{a}}^{2+}+\text{HC}{\text{O}}_{3}^{-}\to \text{CaC}{\text{O}}_{3}+{\text{H}}^{+} \end{array}$$

It is possible that *Achromatium* generates ATP across calcite-containing vacuole membranes containing V-type ATPases and PPase, by analogy to ATP generation across vacuolar membranes containing nitrate in “*Candidatus* Allobeggiatoa” (Beutler et al., 2012). If this process occurs, it represents a previously unrecognized mechanism for prokaryotic energy generation based on chemiosmotic gradients produced by mineral precipitation.

In order to evaluate our new hypothesis, we asked whether the calcite precipitation reaction could generate enough energy for cell survival or growth. The amount of calcium in an average *Achromatium* cell is approximately 0.05 nmol Ca/cell (Head et al., 2000b). Ignoring small contributions from free intracellular calcium (<1 fmol Ca) and assuming that all calcium in the cell is in the form of calcite, an average *Achromatium* cell thus contains 0.05 nmol calcite/cell. One mole of precipitated calcite generates 1 mole H^+^ and 1 mole of ATP requires approximately 3 moles of H^+^ under aerobic conditions (Madigan et al., 2006). Therefore, 16 pmol ATP can be generated from the calcite present in an average cell. This exceeds the amount of energy required for the creation of a new *Escherichia coli* cell (one cycle of cell division) by two orders of magnitude under anaerobic or aerobic growth (0.1–0.2 pmol; Hempfling and Mainzer, 1975; Farmer and Jones, 1976; Stouthammer and Bettenhausen, 1977). Assuming that similar energy requirements apply to *Achromatium*, one round of calcite precipitation in a single cell would enable six to seven cycles of cell division, producing a population size of 64–128 *Achromatium* cells.

If cells need to expend energy to pump calcium into vacuoles in order to precipitate calcite, the energy yield from calcite precipitation would be significantly smaller than the estimates above. We also note that ATP generated from calcite precipitation is small compared to ATP that can be generated by reducing nitrate in nitrate-storing vacuoles. Assuming that nitrate vacuoles make up 80% of cell volume, that the vacuoles contain 250 mM nitrate, and that the energy yield is approximately 16 ATP per mole of nitrate (coupled to sulfur oxidation), a typical cell gains between 0.08 and 375 μmol of ATP from the reduction of stored nitrate. This yields at least four orders of magnitude more energy than calcite precipitation. No nitrate vacuoles have been observed in *Achromatium* to date. Instead all known *Achromatium* populations precipitate intracellular calcite, even in waters undersaturated with respect to calcite such as acidic Lake Fuchskuhle (Glöckner et al., 1999) and the freshwater Rydal Water (Gray and Head, 2014). Thus, it remains unclear why *Achromatium* would precipitate calcite to produce energy instead of employing a more energetically rich nitrate storage strategy.

Further work is required to resolve the striking and enigmatic calcite precipitation behavior of *Achromatium* populations. If calcite precipitation serves as an alternative energy generation scheme mediated by V-type ATPases and PPase in vacuolar membranes, calcite inclusions should accumulate when redox energy substrate availability is low. If calcite inclusions function as a reservoir of buffer to offset sulfuric acid production, then they should accumulate in parallel with S(0) inclusions. Given the spatially and temporally dynamic habitats favored by *Achromatium* populations, these two predictions are not mutually exclusive. A third hypothesis, that calcite serves as ballast to anchor *Achromatium* cells vulnerable to being swept away at the sediment water interface, is consistent with existing observations and should also be considered.

### Conflict of interest statement

The authors declare that the research was conducted in the absence of any commercial or financial relationships that could be construed as a potential conflict of interest.
