# Supplementary material for: Metabolic diversity and ecological niches of Achromatium populations revealed with single-cell genomic sequencing
Source: Front Microbiol. 2015 Aug 10;6:822. doi: 10.3389/fmicb.2015.00822 (PMC4530308; doi:10.3389/fmicb.2015.00822)
Supplement: Supplementary file 1 [file Table1.DOCX]

***Supplementary Material***

**Metabolic diversity and ecological niches of *Achromatium* populations revealed with single-cell genomic sequencing**

**Muammar Mansor^1,^*, Trinity L. Hamilton^2^, Matthew S. Fantle^1^, Jennifer L. Macalady^1,^***

^1^Geosciences Department, Pennsylvania State University, University Park, Pennsylvania, USA

^2^Department of Biological Sciences, University of Cincinnati, Cincinnati, Ohio, USA

***Correspondence:** Muammar Mansor or Jennifer Macalady, Geosciences Department, Pennsylvania State University, University Park, Pennsylvania, 16803, USA. muammar10@gmail.com, jlm80@psu.edu

1. **Supplementary Tables**

Supplementary Table 1: List of gene markers used to estimate genome completeness.

| **Gene markers** | **Presence** | | |
| --- | --- | --- | --- |
|  | **WMS1** | **WMS2** | **WMS3** |
| **rRNAs** |  |  |  |
| 23S | + | + | + |
| 16S | + | + | + |
| 5S | - | + | + |
| **tRNA synthetases** |  |  |  |
| Ala | + | + | + |
| Arg | + | + | + |
| Asn | + | + | + |
| Asp | + | + | + |
| Cys | + | + | + |
| Gln | + | + | + |
| Glu | + | + | + |
| Gly | + | + | + |
| His | + | + | + |
| Ile | + | + | + |
| Leu | + | + | + |
| Lys | + | + | + |
| Met | - | + | + |
| Phe | - | + | + |
| Pro | + | + | + |
| Ser | + | + | + |
| Thr | - | + | + |
| Trp | + | + | + |
| Try | + | + | + |
| Val | + | + | + |
| **DNA maintenance** |  |  |  |
| DNA pol III, α subunit | - | + | + |
| DNA pol III, β subunit | - | - | + |
| DNA pol III, ε subunit | + | + | + |
| DNA pol III, γ and τ subunit | + | + | + |
| DNA pol III, δ subunit | - | + | + |
| DNA pol III, δ' subunit | + | - | + |
| Replicative DNA helicase | - | + | + |
| DNA primase | + | + | + |
| DNA gyrase, A subunit | + | + | + |
| DNA gyrase, B subunit | + | + | + |
| DNA ligase | - | + | - |
| SSB | - | - | + |
| DNA pol I | + | + | - |
| Ribonuclease H | - | + | - |
| Endonuclease III | + | + | + |
| Uracil-DNA glycosylase | + | + | + |
| **RNA transcription** |  |  |  |
| RNA helicase | - | + | + |
| Transcription elongation factor *greA* | - | - | + |
| Transcription termination protein *NusA* | - | + | + |
| RNA polymerase, β subunit | - | + | + |
| RNA polymerase, β' subunit | - | + | + |
| RNA polymerase, α subunit | - | + | + |
| RNA polymerase, ω subunit | - | + | - |
| σ54 factor RpoN | + | + | + |
| σ factor RpoS | - | + | + |
| σ factor RpoD | - | - | + |
| σ factor RpoE | - | + | + |
| σ factor RpoH | + | + | + |
| **RNA translation** |  |  |  |
| Cysteine desulfurase | - | + | + |
| Ribonuclease PH | - | + | + |
| Peptidyl-tRNA hydrolase | + | + | + |
| Elongation factor P | + | + | + |
| Elongation factor G | - | + | - |
| N(5)-glutamine methyltransferase *PrmC* | + | + | + |
| N(5)-glutamine methyltransferase *PrmB* | + | + | + |
| Initiation factor 1 | + | + | + |
| Initiation factor 2 | - | + | + |
| Initiation factor 3 | - | + | + |
| Elongation factor *LepA* | + | + | + |
| Peptide chain release factor 1 | + | + | + |
| tmRNA-binding protein *SmpB* | - | + | + |
| Elongation factor Ts | - | + | + |
| Elongation factor Tu | + | + | + |
| Polyribonucleotide nucleotidyltransferase | + | + | - |
| Ribonuclease III | + | + | + |
| Ribonuclease P | - | + | + |
| **Ribosomal proteins** |  |  |  |
| L1 | + | + | + |
| L2 | - | - | + |
| L3 | - | + | + |
| L4 | - | - | + |
| L5 | - | - | - |
| L6 | - | - | - |
| L7/L12 | - | + | + |
| L9 | - | + | + |
| L10 | - | + | + |
| L11 | + | + | + |
| L13 | - | + | + |
| L14 | - | - | - |
| L15 | - | - | - |
| L16 | - | - | - |
| L17 | - | + | + |
| L18 | - | - | - |
| L19 | - | + | + |
| L20 | - | + | + |
| L21 | + | + | - |
| L22 | - | - | + |
| L23 | - | - | - |
| L24 | - | - | - |
| L25 | + | + | - |
| L26/S20 | - | + | + |
| L27 | + | + | - |
| L28 | + | + | + |
| L29 | - | - | - |
| L30 | - | - | - |
| L31 | - | + | + |
| L32 | - | + | - |
| L33 | + | + | + |
| L34 | - | + | + |
| L35 | - | + | + |
| L36 | - | - | - |
| S1 | - | + | - |
| S2 | - | + | + |
| S3 | - | - | - |
| S4 | - | + | - |
| S5 | - | - | - |
| S6 | + | + | + |
| S7 | - | - | + |
| S8 | - | - | - |
| S9 | - | + | + |
| S10 | - | + | + |
| S11 | - | + | - |
| S12 | - | + | + |
| S13 | - | + | - |
| S14 | - | - | - |
| S15 | - | + | - |
| S16 | - | + | + |
| S17 | - | - | - |
| S18 | + | + | - |
| S19 | - | - | + |
| S21 | + | + | + |
| **Protein processing** |  |  |  |
| Methionine aminopeptidase | - | + | + |
| Aminopeptidase *pepA* | - | + | + |
| Chaperone protein *dnaK* | + | + | + |
| Chaperone protein *dnaJ* | + | + | + |
| Hsp60 *groES* | - | - | + |
| Hsp60 groEL | - | + | + |
| Hsp *grpE* | + | + | + |
| Signal recognition particle subunit *ffh* | + | + | + |
| Signal recognition particle receptor protein *ftsY* | - | + | + |
| Preprotein translocase subunit *secE* | + | + | + |
| Preprotein translocase subunit *secD/F* | - | + | + |
| Preprotein translocase subunit *secY* | - | + | - |
| Preprotein translocase subunit *yajC* | - | + | + |
| Preprotein translocase subunit *yidC* | - | - | + |
| Preprotein translocase subunit secA | - | + | + |
| Preprotein translocase subunit secB | - | + | - |
| ATP-dependent protease HsIV | + | + | + |
| ATP-dependent protease La | - | + | + |
| Cell division protein *ftsZ* | + | - | + |
| Cell division protein ftsW | - | + | + |
| **F-type ATPase** |  |  |  |
| ATP synthase α chain | - | - | + |
| ATP synthase a chain | - | + | + |
| ATP synthase ε chain | - | + | - |
| ATP synthase β chain | - | + | + |
| ATP synthase c chain | - | - | + |
| ATP synthase b chain | - | - | + |
| ATP synthase γ chain | - | + | + |
| ATP synthase δ chain | - | - | + |
| **V-type ATPase** |  |  |  |
| A subunit | - | + | + |
| B subunit | - | + | + |
| C subunit | - | + | - |
| D subunit | - | - | + |
| E subunit | + | + | - |
| I subunit | + | + | + |
| K subunit | + | + | + |
