# Supplementary material for: Metabolic diversity and ecological niches of Achromatium populations revealed with single-cell genomic sequencing
Source: Front Microbiol. 2015 Aug 10;6:822. doi: 10.3389/fmicb.2015.00822 (PMC4530308; doi:10.3389/fmicb.2015.00822)
Supplement: Supplementary file 2 [file Table2.DOCX]

***Supplementary Material***

**Metabolic diversity and ecological niches of *Achromatium* populations revealed with single-cell genomic sequencing**

**Muammar Mansor^1,^*, Trinity L. Hamilton^2^, Matthew S. Fantle^1^, Jennifer L. Macalady^1,^***

^1^Geosciences Department, Pennsylvania State University, University Park, Pennsylvania, USA

^2^Department of Biological Sciences, University of Cincinnati, Cincinnati, Ohio, USA

***Correspondence:** Muammar Mansor or Jennifer Macalady, Geosciences Department, Pennsylvania State University, University Park, Pennsylvania, 16803, USA. muammar10@gmail.com, jlm80@psu.edu

1. **Supplementary Tables**

Supplementary Table 2: List of identified metabolic genes in the draft genomes of *Achromatium*

| **Genes** | **Draft genome** | | |
| --- | --- | --- | --- |
|  | **WMS1** | **WMS2** | **WMS3** |
| Oxygen metabolism |  |  |  |
| Cytochrome c oxidase (*coxABC*) | B | AC | ABC |
| Carbon metabolism |  |  |  |
| Acetate permease | - | + | + |
| Acetyl-CoA synthetase (ACS) | - | + | + |
| Carbonic anhydrase | + | + | + |
| RuBisCO (*rbcLS*) | - | LS | LS |
| Polyhydroxyalkanoate (PHA) synthase | + | - | + |
| Glycogen synthase | - | - | + |
| Nitrogen metabolism |  |  |  |
| Nitrogenase (*nifDHK*) | - | DHK | DHK |
| Copper-containing nitrite reductase (EC 1.7.2.1) | - | + | - |
| NO reductase (*norBC*) | BC | BC | BC |
| Putative nitrate reductase (hydroxylamine reductase) | - | - | + |
| Sulfur oxidation/reduction |  |  |  |
| Sulfide:quinone reductase (*sqr*) | - | Type C | Type A |
| *sox* |  |  |  |
| ABXYZ | - | ABYZ | AZ |
| CD | D | - | - |
| Reverse dissimilatory sulfite reductase (*rdsrAB*) | - | AB | B |
| *dsrMKJOP* | - | JP | MKJOP |
| Sulfate adenyltransferase (*sat*) | + | + | + |
| Adenosine-5′-phosphosulfate reductase (*aprAB*) | - | + | + |
| Polysulfide reductase (NrfD-like) | - | + | + |
| Rhodanese | - | + | + |
| Thiosulfate reductase (*phsABC*) | - | C | - |
| Heterodisulfide reductase (*hdrABC*) | ABC | ABC | - |
| Sulfur globule proteins (*sgpABC*) | - | B | B |
| Hydrogen metabolism |  |  |  |
| Isp type 1 [NiFe]-hydrogenase (large and small subunits) | - | + | - |
| [FeFe] hydrogenase, homology to coenzyme F420 oxidoreductase | + | - | + |
| [FeFe] hydrogenase | - | + | + |
| Motility-related |  |  |  |
| Twitching motility gene set (*pilGHIJTU*) | G | GHIJT | GHIJTU |
| Chemotaxis response genes/protein |  |  |  |
| Aerotaxis receptor (*aer*) | - | - | + |
| Methyl chemotaxis proteins (MCP) | - | + | + |
| *cheABRWY* | ABRY | ABWY | ABRWY |
| Motility protein B (*motB*) | - | + | + |
| Transporters |  |  |  |
| Ca^2+^ ATPase (PMCA-like) | - | + | + |
| YrbG / Cation:H^+^ antiporter / Na:Ca exchanger (*yrbG*) | - | + | + |
| V-type ATPase (ABCDEIK subunits) | EIK | ABCEIK | ABDIK |
| Pyrophospate-energized H^+^/Na^+^/K^+^ pump (*hppA*) | + | + | + |
| Arsenate metabolism |  |  |  |
| Arsenate reductase (*arsC*) | - | + | - |
| Phosphate metabolism |  |  |  |
| Polyphosphate kinase (*ppk*) | + | + | + |
| Siderophore-related |  |  |  |
| Siderophore-specific ABC transporter, periplasmic component | + | + | - |
| Siderophore transport system, periplasmic protein (*tonB*) | - | + | - |
| Siderophore transport system, transport protein (*exbB*) | - | - | + |
| TonB-dependent copper receptor | - | + | + |
| Antibiotic resistance |  |  |  |
| Vancomycin resistance protein (*vanW*) | - | - | + |
| Multidrug transporter (*tolC*) | - | - | + |
