# Supplementary figures and images for: Metabolic diversity and ecological niches of Achromatium populations revealed with single-cell genomic sequencing
Source: Front Microbiol. 2015 Aug 10;6:822. doi: 10.3389/fmicb.2015.00822 (PMC4530308; doi:10.3389/fmicb.2015.00822)

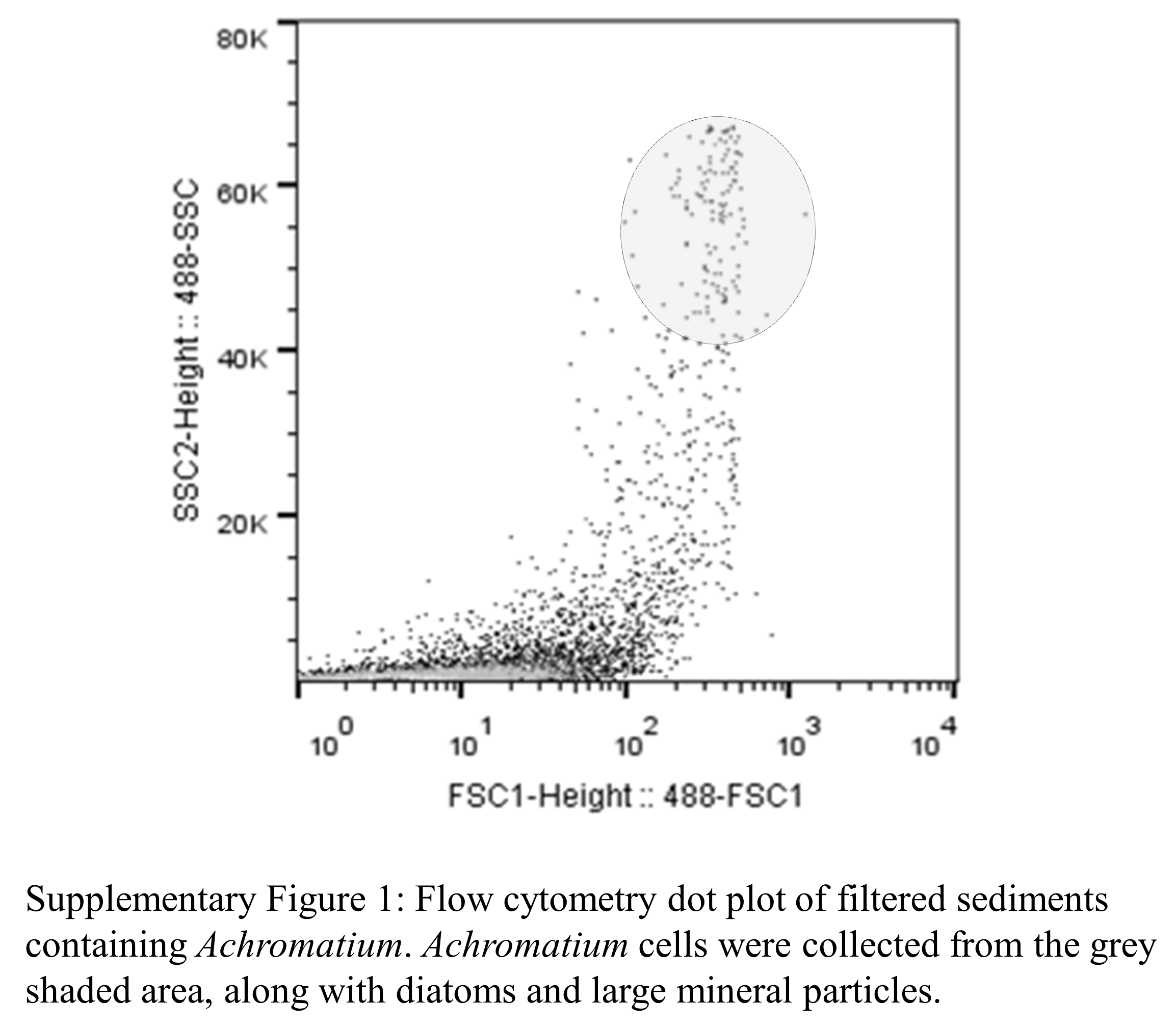

Supplement: Supplementary file 3 [file Image1.TIF]

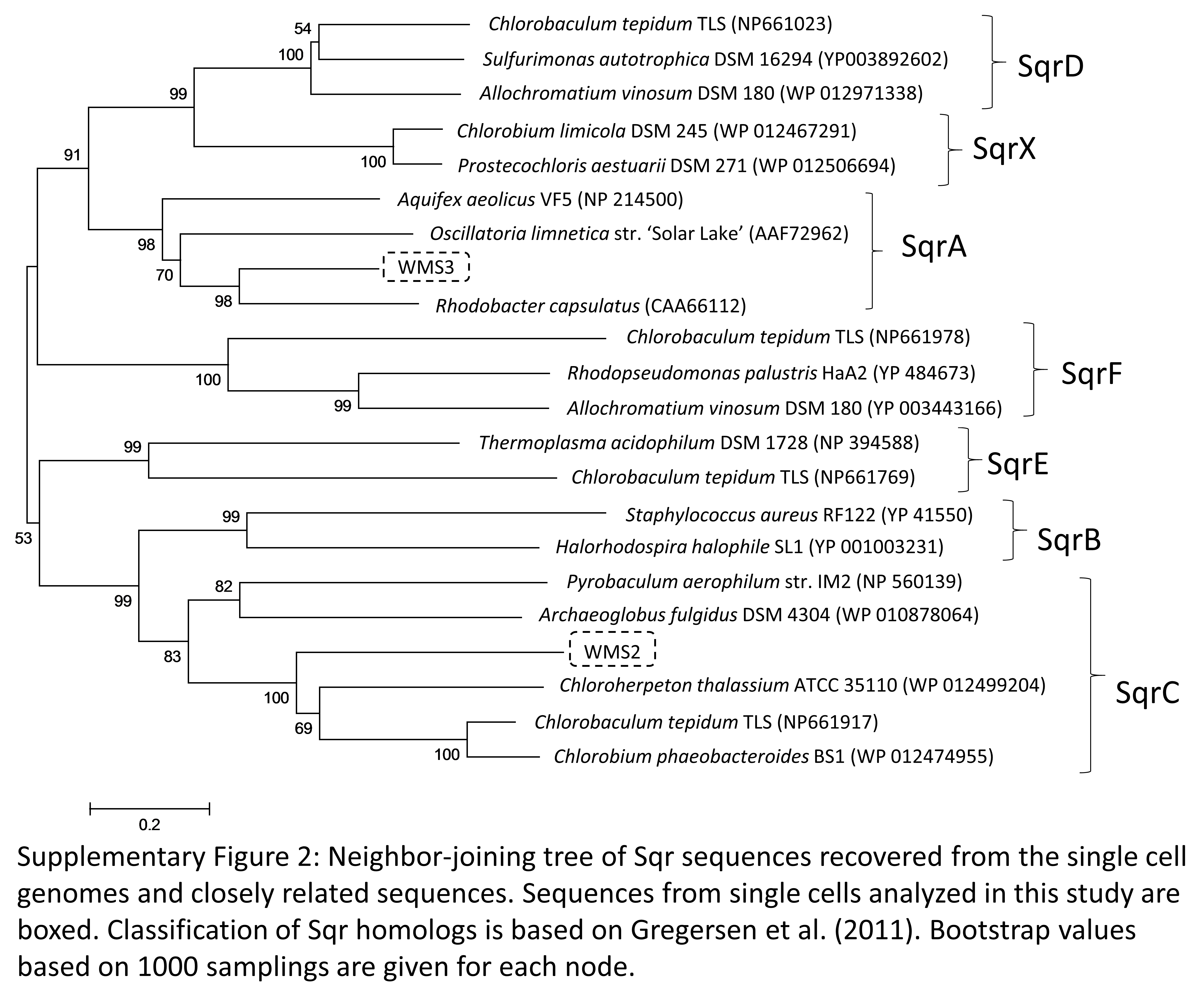

Supplement: Supplementary file 4 [file Image2.TIF]

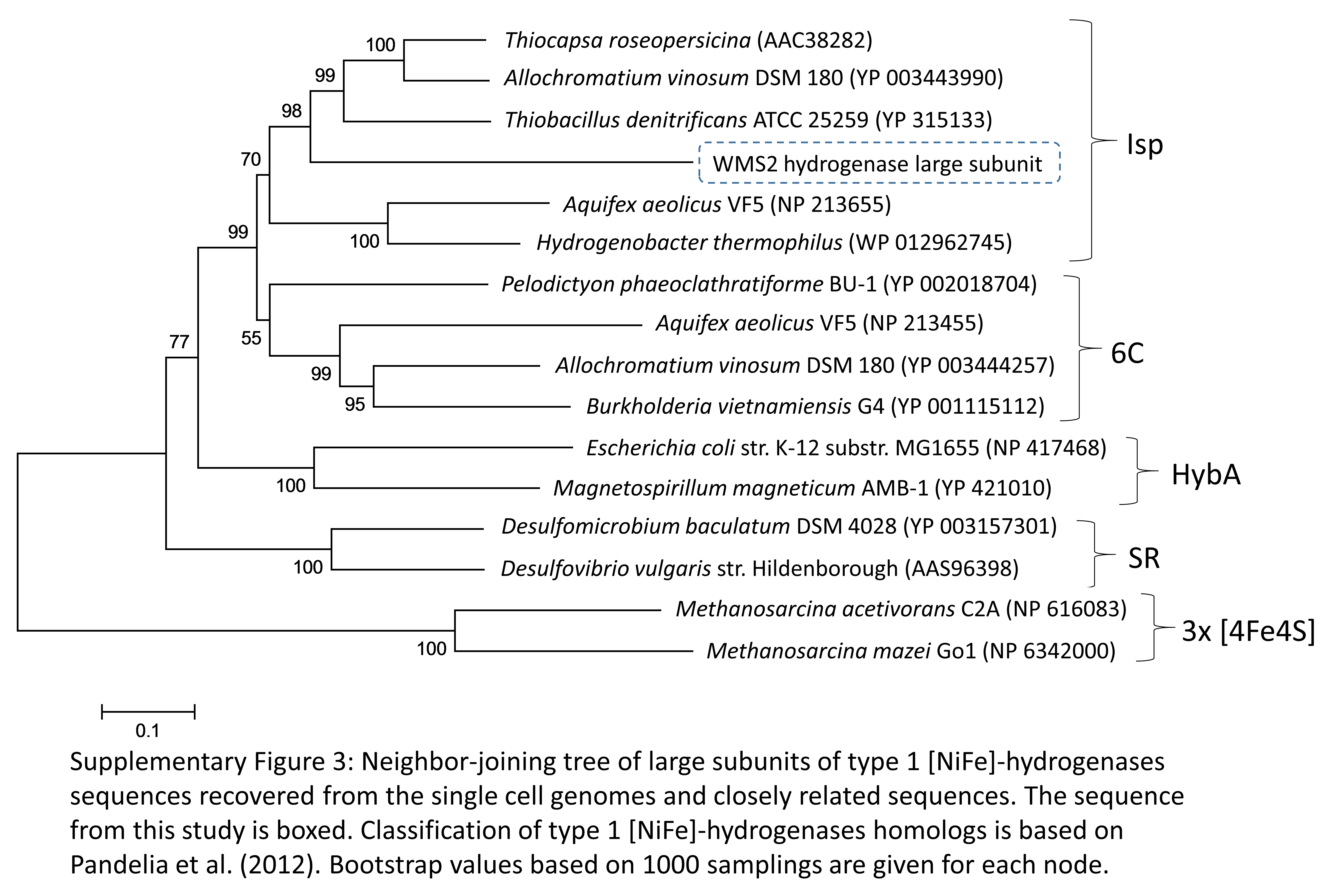

Supplement: Supplementary file 5 [file Image3.TIF]
